# KDM4B Knockdown Attenuates Neurological Deficits in Ischemic Stroke by Inhibiting Neuronal Apoptosis Associated With Birc5 Upregulation

**DOI:** 10.1002/cns.71264

**Published:** 2026-11-11

**Authors:** Siyu Deng, Nueraili Maihemuti, Mengjie Jia, Keli Huang, Mao Xie, Qi He, Jing Zhao

**Affiliations:** ^1^ Department of Pathophysiology Chongqing Medical University Chongqing People's Republic of China; ^2^ Center for Neuroscience Research Chongqing Medical University Chongqing People's Republic of China; ^3^ Department of Pathology Chongqing Medical University Chongqing People's Republic of China; ^4^ Department of Critical Care Medicine The First Affiliated Hospital of Chongqing Medical University Chongqing China

**Keywords:** apoptosis, Birc5, caspase cascade, ischemic stroke, KDM4B

## Abstract

**Background:**

KDM4B (Lysine demethylase 4B), a histone lysine demethylase, exerts a crucial epigenetic regulatory role in the progression of numerous disorders by catalyzing demethylation reactions on target proteins. However, the precise biological function and molecular mechanism by which KDM4B regulates neurological deficits in ischemic stroke remains elusive.

**Methods:**

In this study, we employed a mouse middle cerebral artery occlusion/reperfusion (MCAO/R) model and an oxygen–glucose deprivation/reoxygenation (OGD/R) model in HT22 neurons to mimic ischemic stroke. Targeted loss‐of‐function approaches were executed utilizing in vivo siRNA delivery and in vitro lentiviral shRNA transduction. The neuroprotective effects were systematically evaluated through infarct volume quantification, histopathological staining, and diverse sensorimotor behavioral tests. Furthermore, transcriptomic profiling (RNA‐seq) coupled with molecular biology assays was performed to elucidate the downstream apoptotic cascades and key targets governed by KDM4B.

**Results:**

Herein, we found that KDM4B is predominantly localized in neurons, with its expression significantly decreasing initially and then gradually increasing following ischemic stroke. Loss‐of‐function experiments demonstrated that KDM4B knockdown mitigated brain damage, attenuated tissue structure disruption, and improved sensorimotor dysfunctions in mice with ischemic stroke. Additionally, KDM4B knockdown protected neurons against OGD/R insult. Furthermore, KDM4B knockdown inhibited the expression of Bax and the activation of the pro‐apoptotic caspase cascade (caspase‐3/6/7/8/9), while upregulating the expression of the anti‐apoptotic protein Bcl‐2, thereby reducing neuronal apoptosis. Mechanistically, KDM4B knockdown is associated with an upregulation of Birc5 expression, which in turn inhibits apoptotic progression in neurons.

**Conclusions:**

These results demonstrate that KDM4B knockdown alleviates neurological deficits following ischemic stroke by inhibiting neuronal apoptosis, partially accompanied by Birc5.

## Graphical abstract

In ischemic stroke, knockdown of the histone demethylase KDM4B was associated with Birc5 upregulation, a key member of the inhibitor of apoptosis protein (IAP) family. This was further associated with increased Bcl‐2 expression, decreased Bax expression, and reduced activation of initiator caspases (caspase‐8/9) and effector caspases (caspase‐3/6/7), ultimately correlating with attenuated neuronal apoptosis and improved neurological deficits.

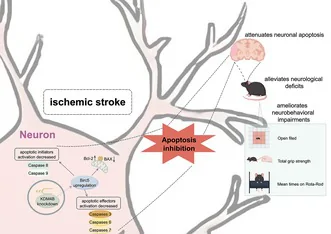

## 1 Introduction

Ischemic stroke is predominantly caused by cerebral artery occlusion, which interrupts the supply of glucose and oxygen to the brain and ultimately leads to brain injury. As a major global health threat, ischemic stroke accounts for a substantial proportion of deaths and disabilities worldwide, imposing a huge economic burden on society [1, 2]. Although thrombolytic therapy and mechanical thrombectomy can effectively recanalize occluded arteries, there remains a lack of effective strategies to protect the infarct core and salvage the surrounding ischemic penumbra [3]. Consequently, a large number of ischemic stroke patients—whether treated clinically or not—still experience long‐term cognitive and psychomotor impairments. Therefore, in‐depth research into therapeutic strategies for ischemic stroke is a global priority.

Apoptosis, a fundamental biological process, is pivotal to the pathogenesis of numerous disorders, including ischemic stroke. Apoptosis is genetically orchestrated primarily via two core pathways: the intrinsic pathway, initiated by mitochondrial dysfunction, and the extrinsic pathway, mediated by extracellular signals [4]. During ischemic stroke, mitochondrial dysfunction in neurons of the ischemic penumbra induces the opening of the mitochondrial translocation pore, which releases pro‐apoptotic factors and subsequently triggers intrinsic apoptotic cascades. Concurrently, death ligands—including TNF‐α (tumor necrosis factor‐α) and FasL (Fas ligand)—secreted by infiltrating immune cells and dying cells within the infarct area bind to death receptors on the surface of ischemic penumbral neurons, thereby activating the extrinsic apoptotic signaling cascade. Thus, apoptosis stands out as the predominant form of programmed neuronal death and contributes significantly to irreversible neurological damage following ischemic stroke [5, 6]. Identifying key molecules that modulate apoptotic pathways and inhibiting neuronal apoptosis in the ischemic penumbra represents promising therapeutic strategies for ischemic stroke.

KDM4B is usually localized in the nucleus and is characterized by the catalytic Jumonji‐C domain. KDM4B is believed to regulate the transcriptional expression of target genes by catalyzing demethylation at specific lysine residues within histone tails [7]. Additionally, KDM4B can interact with a subset of non‐histone proteins, such as TRAF6 (TNF receptor‐associated Factor 6) and c‐MYC (cellular MYC proto‐oncogene), to modulate their biological activities in a demethylase activity‐dependent manner [8, 9]. Accumulating studies have demonstrated that KDM4B exerts a crucial epigenetic regulatory role in the tumorigenesis of numerous malignancies by modulating cell differentiation, proliferation, metastasis, and drug resistance, thus emerging as a potential therapeutic target [10]. Additionally, growing evidence indicates that KDM4B also plays an important regulatory role in aging, obesity, and metabolic disorders [11, 12, 13]. However, little is currently known about the function of KDM4B on neurological disorders, especially ischemic stroke. In the present study, we sought to investigate the functional role of KDM4B in neurological impairment following ischemic stroke and to elucidate the underlying phenotypic mechanisms from the perspective of cellular apoptosis.

## 2 Materials and Methods

This part of the content is provided in detail in Supporting Information [14, 15, 16]. The primer sequences used in the qPCR assay are provided in Table 1. Detailed antibody information is provided in Table 2.

**TABLE 1:** Primer sequences used in qPCR assay.

Gene	Forward primer sequence (5′‐3′)	Reverse primer sequence (5′‐3′)
NFKBIA	ACCTGGTTTCGCTCTTGTTGAAA	GCCTCCAAACACACAGTCATCAT
Itpr3	AGCACATCAAACTGGAGCATAAC	CCAGTCCAGGTTCTTGTTCTTGA
Kdm4b	TCGCCCATCATCCTGAAGAAGTA	TTAGTGGACTCAGCGCAGTTAAA
Ptpn13	AGGCAGTCAGAGTATTGGTTGTT	CCAGAATCCGCCCAAGAACTAAT
Ctsh	GGAAATGTTGTTTCGCCAGTGAA	GGGTAGCTGTCTTCTTCCATGAT
Fos	GGAGAATCCGAAGGGAACGG	GCAATCTCAGTCTGCAACGC
Gadd45b	GGCCAAACTGATGAATGTGGAC	CTGGATCAGGGTGAAGTGAATCT
Gadd45g	CTGCAGATCCATTTCACGTTGAT	AGGATTCGAAATGAGGATGCAATG
Ngf	CCAAGGACGCAGCTTTCTATACT	GTGTCAAGGGAATGCTGAAGTTT
Map3k14	GAACAAATCCTCTCCTGCCTCAG	GAAGTAGCTCGGGATGTCAGTAG
Septin4	AGCCTCTTCCTCACTGACTTGTA	AGCCTCACTCCCTTCTCTTCTAT
Traf1	AGCCTCTTCTCTCCAGCTTTCTA	CTCTCGGTTGTTCTGGTCAAGTA
Tnf	GCCTCCCTCTCATCAGTTCTATG	ACCTGGGAGTAGACAAGGTACAA
Naif1	TGTACTGCTGACCCCTATGC	TGTGGTAGGTGGTCTCGGT
Birc5	GAATCCTGCGTTTGAGTCGTCTT	TCCCAGCCTTCCAATTCCTTAAA
Birc3	CCTTACAAGCAAGCACACTGATT	CCTCTCGGTCCATACACACTTTA
β‐Actin	AGTGTGACGTTGACATCCGT	GCAGCTCAGTAACAGTCCGC

*Note:* The primer sequences of housekeeping genes in the apoptotic pathway identified by KEGG enrichment analysis (including Nfkbia, Itpr3, Kdm4b, Ptpn13, Ctsh, Fos, Gadd45b, Gadd45g, Ngf, Map3k14, Septin4, Traf1, Tnf, Naif1, Birc5 and Birc3) used in the qPCR assay are listed in Table 1.

**TABLE 2:** Antibody information.

Antibody	Company	Item number	Application (dilution ratio)
Anti‐KDM4B	Invitrogen	MA5‐35692	Immunofluorescence staining (1:100)
Anti‐NeuN	Invitrogen	MA5‐33103	Immunofluorescence staining (1:200)
Anti‐GFAP	Invitrogen	14–9892‐82	Immunofluorescence staining (1:200)
Anti‐iba1	Invitrogen	MA5‐27726	Immunofluorescence staining (1:200)
Dylight 488 goat anti‐rabbit IgG	Abbkine	A23220	Immunofluorescence staining (1:200)
Dylight 594 goat anti‐mouse IgG	Abbkine	A23410	Immunofluorescence staining (1:200)
Anti‐KDM4B	Santa Cruz	sc‐374,241	Western blot (1:100)
Anti‐Bax	Proteintech	60,267–1‐Ig	Western blot (1:1000)
Anti‐Bcl‐2	Abclonal	A11025	Western blot (1:1000)
Anti‐Caspase‐3	Cell Signaling Technology	9662	Western blot (1:1000)
Anti‐Caspase‐6	Abclonal	A22437	Western blot (1:1000)
Anti‐Caspase‐7	Cell Signaling Technology	9492	Western blot (1:1000)
Anti‐Caspase‐8	Cell Signaling Technology	4927	Western blot (1:1000)
Anti‐Caspase‐9	Cell Signaling Technology	9504	Western blot (1:1000)
Anti‐ NAIF1	Abclonal	A15217	Western blot (1:1000)
Anti‐Birc3	Proteintech	24,304–1‐AP	Western blot (1:1000)
Anti‐Birc5	Abclonal	A29349	Western blot (1:1000)
Anti‐β‐Actin	Abclonal	AC026	Western blot (1:50000)
HRP‐conjugated secondary antibody (anti‐rabbit)	Invitrogen	31,460	Western blot (1:50000)
HRP‐conjugated secondary antibody (anti‐mouse)	Invitrogen	31,430	Western blot (1:50000)

*Note:* The antibody information used in this research study are listed in Table 2.

## 3 Results

### 3.1 Spatial Expression Profiles of KDM4B in the Ipsilateral Cortex After Cerebral Infarction

To assess the potential involvement of KDM4B in the pathogenesis of ischemic stroke, we established an experimental animal model based on MCAO/R surgery in mice. The success of the MCAO/R model was validated by the reduction in real‐time laser doppler flowmeter (Figure 1A). Time course examination of KDM4B protein showed that KDM4B was decreased in the ipsilateral cortex as early as 6 and 12 h post MCAO operation and then gradually increased to 72 h reperfusion. Specifically, KDM4B expression peaked at 48 h reperfusion in the ipsilateral cortex (Figure 1B,C). Therefore, 48 h reperfusion after MCAO operation was chosen as the specific time point for the subsequent experiments. Immunofluorescence analysis further corroborated this finding, exhibiting a pronounced enhancement in the intensity of KDM4B fluorescence in the ipsilateral peri‐infarct cortex 48 h post MCAO operation compared to the sham‐operated mice (Figure 1D). Furthermore, double‐labeled immunofluorescence revealed that KDM4B was predominantly co‐localized with neurons instead of astrocytes and microglia within the ipsilateral cortex prior to or post MCAO operation (Figure 1D). Based on this finding, we examined the expression of KDM4B in the mouse HT22 neuronal cells stimulated with OGD/R. The level of KDM4B was first decreased after OGD exposure and then gradually increased, reaching the peak at 48 h reoxygenation (Figure 1E,F), which was consistent with the result in animal experiments. Collectively, these animal and cellular results indicate that KDM4B may contribute to the pathological development of ischemic stroke and be related to the neuronal response.

**FIGURE 1:**
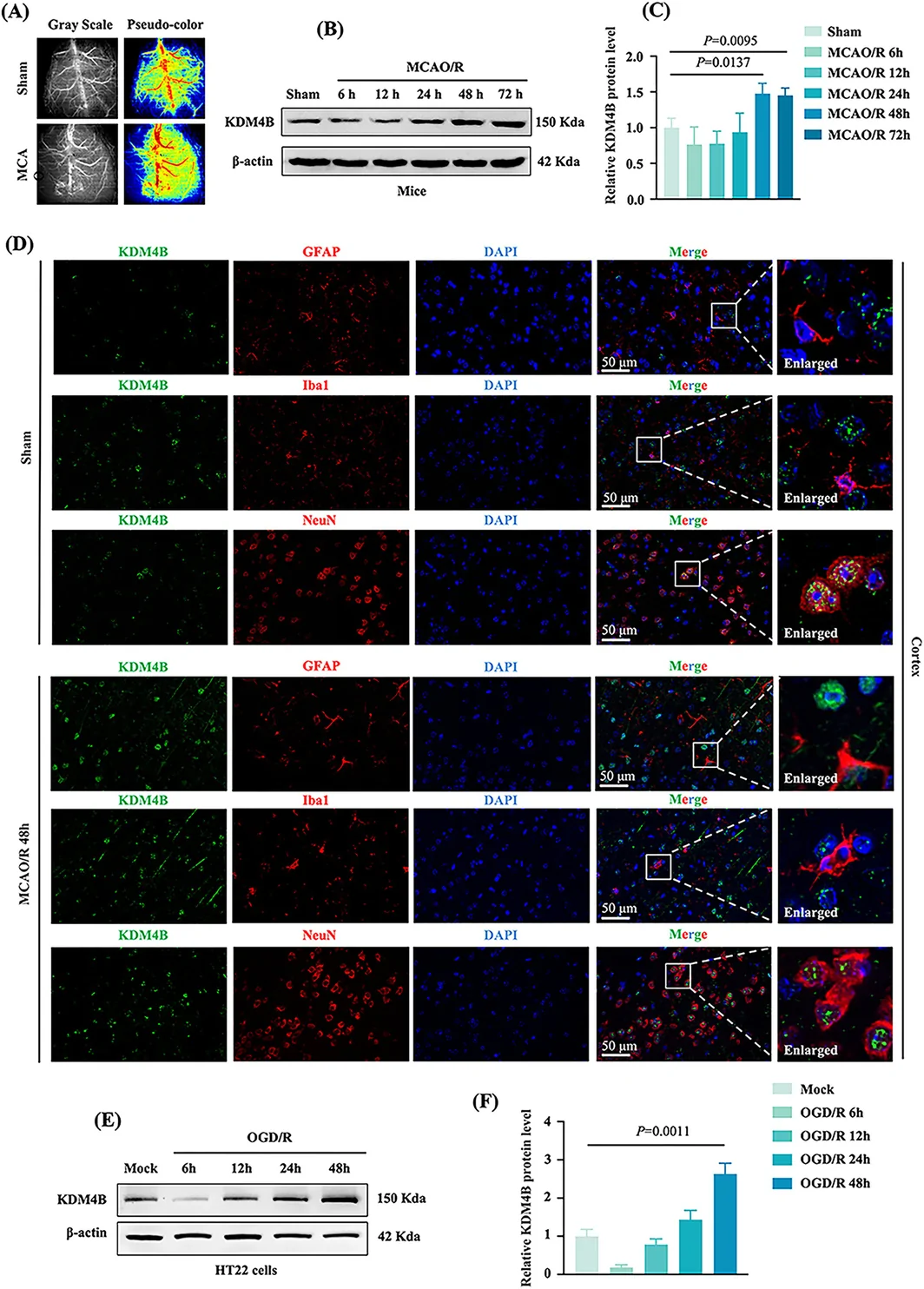
Spatial expression profiles of KDM4B in the ipsilateral cortex after cerebral infarction. (A) Laser Doppler flowmeter for regional cerebral blood flow before and during cerebral ischemia. (B, C) The expression and quantification of the KDM4B protein in mice subjected to 1 h MCAO followed by different reperfusion times, *n* = 3 mice per group. (D) Double‐immunofluorescence analysis of KDM4B with astrocytes (GFAP), microglia (Iba1), or neurons (NeuN) in the cerebral cortex before and after ischemic stroke (scale bars = 50 μm). Single planes labeled with the KDM4B protein showed co‐expression with neurons but not with microglia or astrocytes. The fluorescence intensity of the KDM4B protein in the MCAO/R group was higher than that in the sham group. (E, F) The expression and quantification of the KDM4B protein in HT22 cells exposed to 4 h OGD followed by different reoxygenation times, *n* = 3 independent biological replicates per group. The data represent at least three independent experiments.

### 3.2 KDM4B Knockdown Ameliorated Neurological Impairment in Ischemic Stroke

To determine the biological role of KDM4B in regulating neurological impairment in ischemic stroke, we knocked down KDM4B expression in the MCAO/R mouse models using chemosynthetic siRNAs and detected phenotypic indicators (Figure 2A). The protein level of KDM4B was clearly decreased by different siRNA treatments, and siRNA‐2 exhibited the best interference efficiency in vivo (Figure 2B). Thus, siRNA‐2 was used for KDM4B knockdown in the following experiments. The success of MCAO/R model was also validated by the reduction in real‐time laser doppler flowmeter. For phenotype evaluation, the TTC staining and mNSS were firstly used to estimate brain injury following cerebral ischemia. We observed that the cerebral infarct volumes and neurological deficit scores were significantly decreased after KDM4B knockdown (Figure 2C–E). Then, we performed HE and Nissl staining to assess the tissue structure alteration in the peri‐infarct cortex of ischemic stroke mice. Compared with NC‐siRNA treatment, knockdown of KDM4B prominently improved histological morphology in the peri‐infarct cortex (Figure 2F). Furthermore, we conducted a thorough evaluation of sensorimotor dysfunctions following ischemic stroke via a battery of neurobehavioral tests, including the open field, rotarod, and grip strength tests. Compared with NC‐siRNA treatment, KDM4B knockdown increased the distance of motion and partially restored the exploratory activity in the open field test (Figure 2G,H), extended the time in the rotarod test (Figure 2I), and enhanced the muscle strength in the grip strength test in ischemic stroke mice (Figure 2J). These observations demonstrated a strong protective effect of KDM4B knockdown in ischemic stroke mice.

**FIGURE 2:**
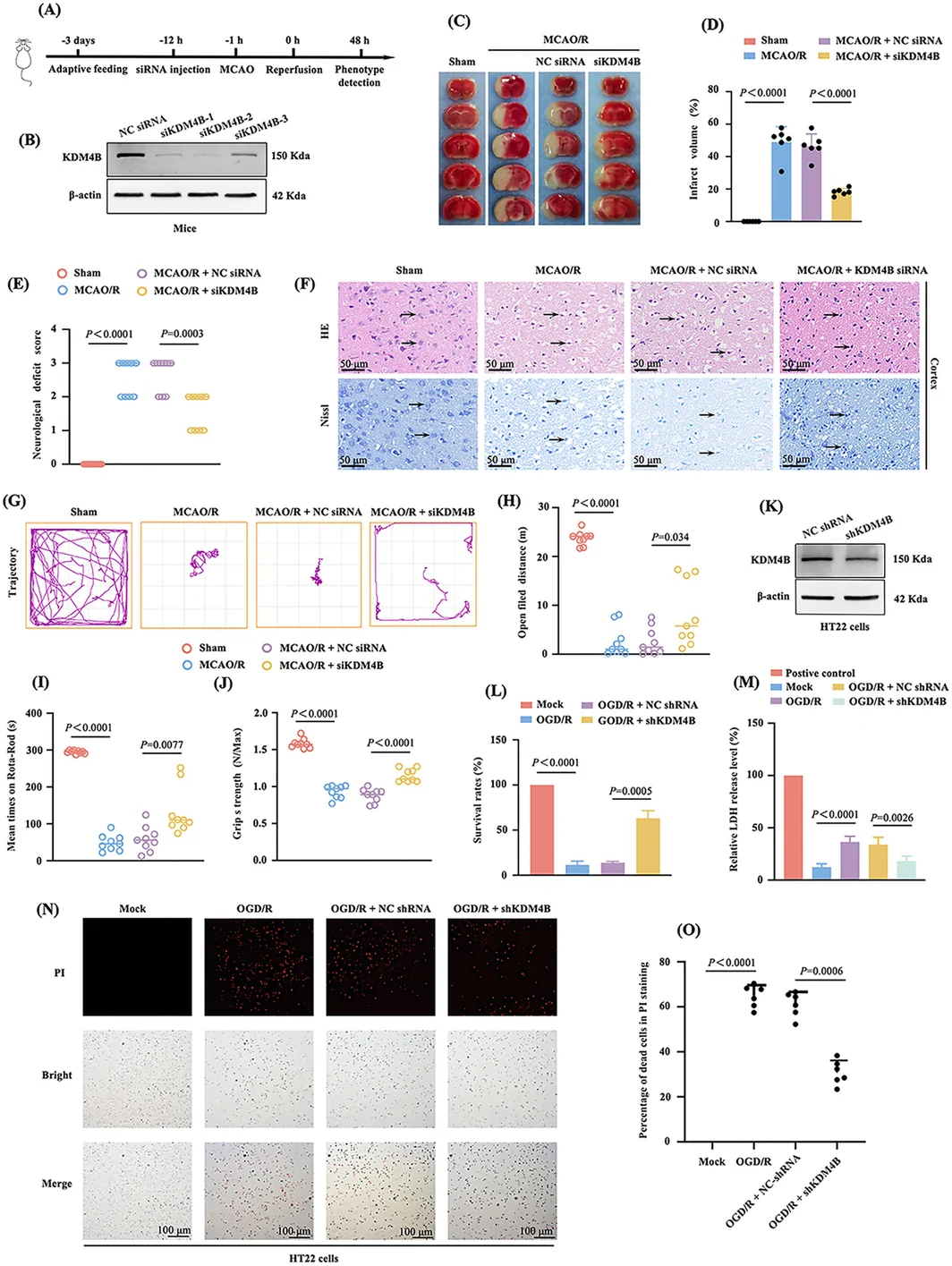
KDM4B knockdown ameliorated neurological impairment in ischemic stroke. (A) Experimental strategy for phenotypic detection in ischemic stroke mice. (B) The protein level of KDM4B in sham mice treated with various chemically synthesized KDM4B‐targeting siRNAs. (C, D) Representative images of coronal brain sections stained with TTC solution and quantification of infarction volume. *n* = 6 mice per group. (E) Neurological deficit scores in ischemic stroke mice treated with NC siRNA or KDM4B siRNA. *n* = 9 mice per group. (F) Representative images of HE and Nissl staining in the cerebral cortex within the ischemic penumbra region of ischemic stroke mice treated with NC siRNA or KDM4B siRNA (Scale bar = 50 μm). *n* = 5 mice per group. (G–J) The open field, rotarod, and grip strength tests for evaluating sensorimotor dysfunction in ischemic stroke mice treated with NC siRNA or KDM4B siRNA. *n* = 9 mice per group. (K) The protein level of KDM4B in HT22 cells stably infected with lentiviral particles encoding KDM4B‐targeting siRNA‐2. (L) The CCK‐8 assay for evaluating viability of HT22 cells stably infected with lentivirus particles encoding NC shRNA or KDM4B shRNA under OGD/R condition. *n* = 12 independent biological replicates per group. (M) The LDH assay for evaluating damage of HT22 cells stably infected with lentivirus particles encoding NC shRNA or KDM4B shRNA under OGD/R condition. *n* = 12 independent biological replicates per group. (N, O) Representative images and quantification for propidium iodide (PI) staining in HT22 cells stably infected with lentivirus particles encoding NC shRNA or KDM4B shRNA under OGD/R condition (Scale bar = 100 μm). Red fluorescence indicates dead HT22 cells. *n* = 5 independent biological replicates per group. The data represent at least three independent experiments.

To explore the influence of KDM4B on neurons in vitro, mouse HT22 neuronal cells were infected with lentiviruses that targeted KDM4B knockdown prior to OGD/R stimulation. The expression of KDM4B in HT22 cells was significantly reduced by lentivirus shKDM4B compared with the control lentivirus (Figure 2K). CCK‐8 and LDH assays revealed that OGD/R treatment significantly reduced the viability and increased the injury of HT22 neuronal cells, respectively. Whereas, KDM4B knockdown reversed these OGD/R‐induced changes in cell viability and injury (Figure 2L,M). In addition, PI staining showed that the death rate was increased in the OGD/R‐treated HT22 neuronal cells. However, the increase in the death rate of HT22 neuronal cells was significantly reversed by KDM4B knockdown (Figure 2N,O).

Taken together, results from animal and cellular phenotypic experiments demonstrated that KDM4B knockdown exerts a neuroprotective effect in ischemic stroke.

### 3.3 Bioinformatics Analysis of KDM4B Functions and Its Regulated Signaling Pathways in Ischemic Stroke

To explore the molecular function of KDM4B in ischemic stroke, we performed mRNA sequencing on ischemic stroke mice with KDM4B knockdown. The transcriptional level of KDM4B was significantly decreased in ischemic stroke mice treated with KDM4B siRNA relative to those receiving NC siRNA, confirming the validity of RNA sequencing analysis (Figure 3A). Volcano plot analysis of differentially expressed gene (DEG) profiles identified 2338 downregulated genes and 963 upregulated genes in KDM4B siRNA‐treated ischemic stroke mice versus NC siRNA‐treated controls (Figure 3B). GO analysis revealed that the biological process changes of these downregulated genes were prominently enriched in the apoptotic process (Figure 3C,D). Molecular function analysis of these downregulated genes demonstrated that cysteine–type endopeptidase regulator activity involved in apoptotic process and cysteine–type endopeptidase inhibitor activity involved in apoptotic process were significantly diminished (Figure 3E). In addition, KEGG enrichment analysis was conducted to further explore the signaling pathways regulated by KDM4B, and the results revealed that these downregulated genes were predominantly enriched in the apoptotic pathway (Figure 3F). Consistently, GSEA enrichment analysis revealed that KDM4B knockdown significantly suppressed the apoptotic pathway (Figure 3G). Overall, these bioinformatics analyses strongly indicate that KDM4B knockdown may suppress apoptosis in ischemic stroke. Given that apoptosis is a key driver of neuronal death and neurological deficits in ischemic stroke, we have reason to believe that KDM4B knockdown alleviates neurological impairment by inhibiting neuronal apoptosis.

**FIGURE 3:**
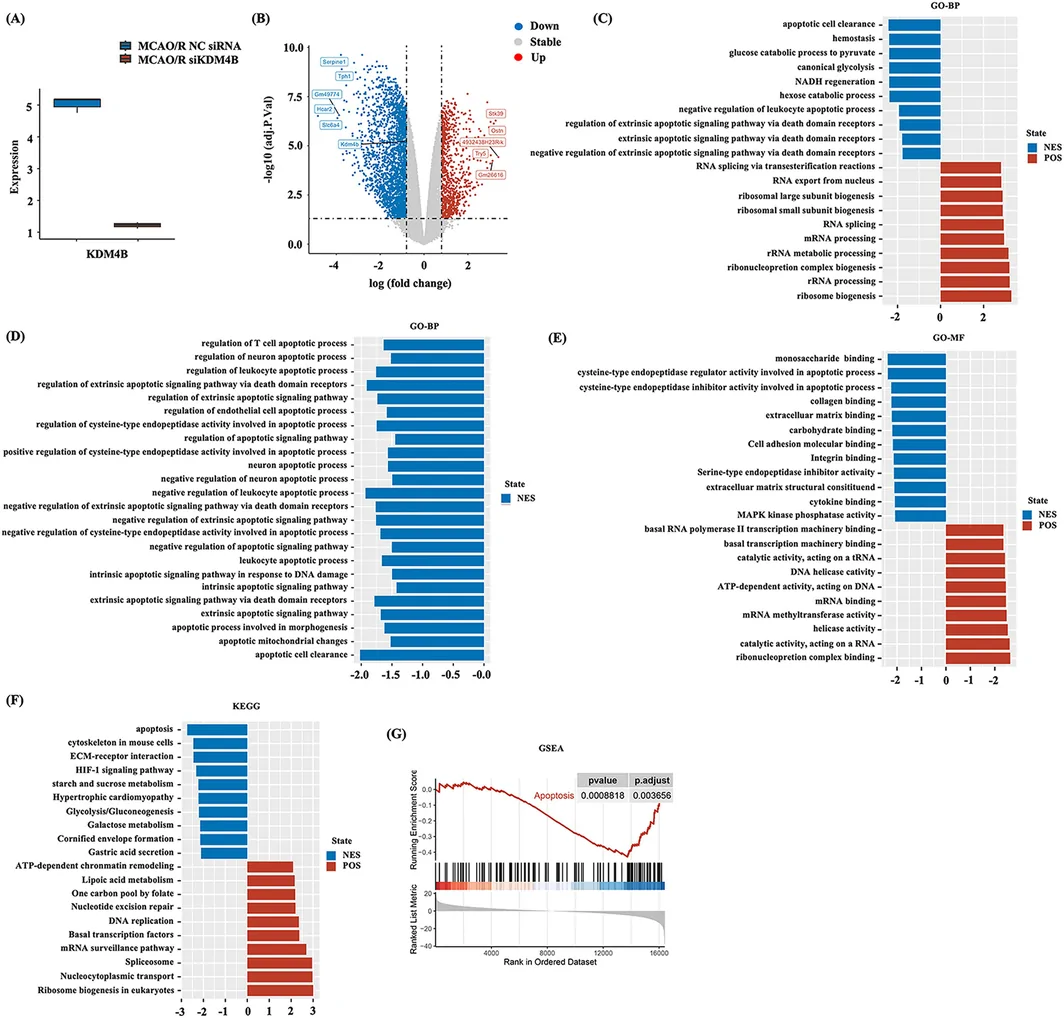
Bioinformatics analysis of KDM4B functions and its regulated signaling pathways in ischemic stroke. Transcriptome analysis was conducted based on the ischemic stroke mice with or without KDM4B knockdown. (A) The expression changes of KDM4B at the transcriptional level in ischemic stroke mice treated with KDM4B‐specific siRNA. *n* = 3 independent biological replicates per group. (B) The volcano plot shows the differentially expressed genes in ischemic stroke mice after KDM4B knockdown; the blue dots represent the downregulated genes, and the red dots represent the upregulated genes. (C) GO analysis of biological processes (BP) for the downregulated and upregulated differentially expressed genes. (D) GO analysis of BP focusing on apoptotic processes. (E) GO analysis of molecular functions (MF) for the downregulated and upregulated differentially expressed genes. (F) KEGG pathway analysis of the downregulated and upregulated genes. (G) GSEA analysis of the apoptosis pathway.

### 3.4 Knockdown of KDM4B Inhibited Neuronal Apoptosis Following Ischemic Stroke

To determine whether KDM4B regulates neurological impairment through neuronal apoptosis, the caspase cascade—an essential executor of apoptosis—was analyzed using western blotting. We found that the activation of the key intrinsic apoptotic initiator caspase 9, the extrinsic apoptotic initiator caspase 8, and the downstream apoptotic effector caspases (including 3, 6, and 7) was enhanced in both the infarcted brains and OGD/R‐stimulated HT22 neuronal cells, whereas KDM4B knockdown caused a significant inhibition of the activation of apoptotic initiator caspases and effector caspases (Figure 4A,B). In addition, we examined the common upstream regulators of the apoptosis process, Bax and Bcl‐2, in the animal and cellular models of ischemic stroke, and found that knocking down KDM4B significantly reduced the level of the pro‐apoptotic proteins Bax and increased the expression of the anti‐apoptotic protein Bcl‐2 (Figure 4A,B). Furthermore, TUNEL staining was performed in the peri‐infarct region of ischemic stroke brain and the OGD/R‐stimulated neuronal HT22 cells. We found that the number of TUNEL‐positive cells was increased by cerebral infarction and OGD/R exposure. However, KDM4B knockdown significantly reduced the proportion of apoptotic cells (Figure 4C–F). Consistently, flow cytometry results showed that the proportion of early and late apoptotic cells was remarkably reduced by KDM4B knockdown in the OGD/R model of HT22 neuronal cells (Figure 4G,H). Overall, the above in vivo and in vitro experimental results not only reveal the inhibitory effect of KDM4B knockdown on the intrinsic and extrinsic apoptotic pathways but also demonstrate its protective effect against neuronal apoptosis after ischemic stroke.

**FIGURE 4:**
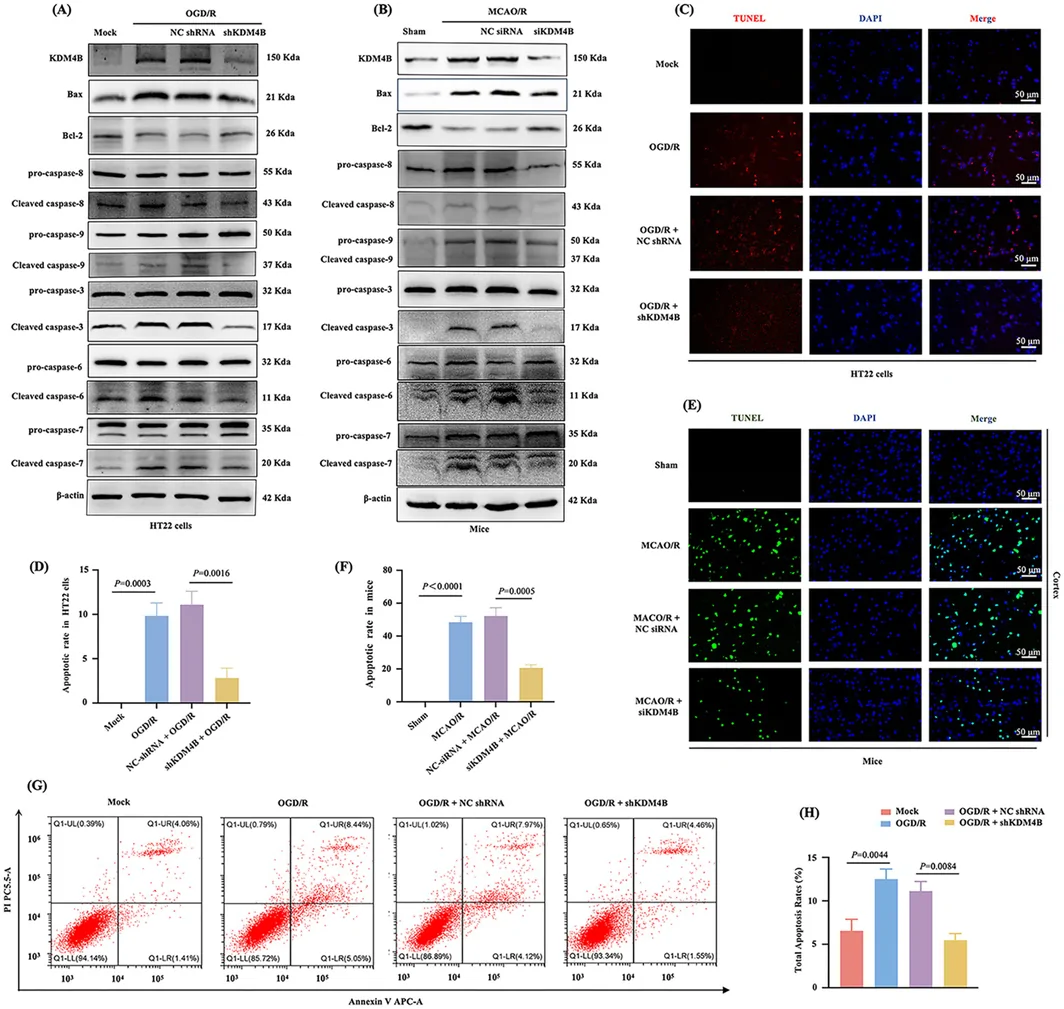
Knockdown of KDM4B inhibited neuronal apoptosis following ischemic stroke. (A) Representative western blotting for the expression of KDM4B and apoptosis regulators (Bax and Bcl‐2) and the activation of apoptotic initiators (caspases‐8 and caspases‐9) and effector caspases (caspases‐3, caspases‐6, and caspases‐7) in HT22 cells with or without KDM4B knockdown under OGD/R condition. *n* = 4 independent biological replicates per group. (B) Representative western blotting for the expression of KDM4B and apoptosis regulators (Bax and Bcl‐2) and the activation of apoptotic initiators (caspases‐8 and caspases‐9) and effector caspases (caspases‐3, caspases‐6, and caspases‐7) in ischemic stroke mice with or without KDM4B knockdown. *n* = 5 mice per group. (C, D) The representative images and statistical quantification of TUNEL staining in HT22 cells with or without KDM4B knockdown under OGD/R condition (Scale bar = 50 μm). *n* = 5 independent biological replicates per group. (E, F) The representative images and statistical quantification of TUNEL staining in the cerebral cortex of ischemic stroke mice with or without KDM4B knockdown (Scale bar = 50 μm). *n* = 5 mice per group. (G, H) The representative flow cytometry scatter plot and statistical analysis of apoptotic HT22 cells with or without KDM4B knockdown under OGD/R condition. *n* = 6 independent biological replicates per group. The data represent at least three independent experiments.

### 3.5 KDM4B Knockdown Reduces Neuronal Apoptosis Associated With Birc5 Upregulation

To explore the mechanisms through which KDM4B knockdown inhibited neuronal apoptosis in ischemic stroke, we further analyzed the significantly differentially expressed component genes of the apoptotic signaling pathway in RNA sequencing data. As shown in the (Figure 5A), 12 obviously downregulated genes and 3 significantly upregulated genes were observed after KDM4B knockdown (Figure 5A). Subsequently, the expression of these 15 differentially expressed component genes in the apoptotic signaling pathway was measured via qPCR in both animal and cellular models of ischemic stroke. We found that these genes exhibited consistent expression trends with the RNA sequencing data after KDM4B knockdown, except for differences in the degree of variation (Figure 5B,C). Of particular interest, three upregulated genes, including NAIF (nuclear apoptosis‐inducing Factor 1), Birc5 (baculoviral inhibitor of apoptosis protein repeat‐containing 5) and Birc3 (baculoviral inhibitor of apoptosis protein repeat‐containing 3) displayed the most dramatic expression alterations in both RNA sequencing and qPCR analyses (Figure 5A–C), suggesting that they may account for the anti‐apoptotic effect of KDM4B knockdown. To verify this possibility, we examined the influence of KDM4B on NAIF, Birc5, and Birc3 in animal and cellular models of ischemic stroke. Our results suggested that KDM4B knockdown selectively upregulated the expression of Birc5, with no significant changes in NAIF or Birc3 levels (Figure 5D–G). Building on previous findings that Birc5 plays a broad and well‐documented negative regulatory role in apoptosis, we conclude that the suppression of neuronal apoptosis by KDM4B knockdown in ischemic stroke is closely linked to the specific upregulation of Birc5.

**FIGURE 5:**
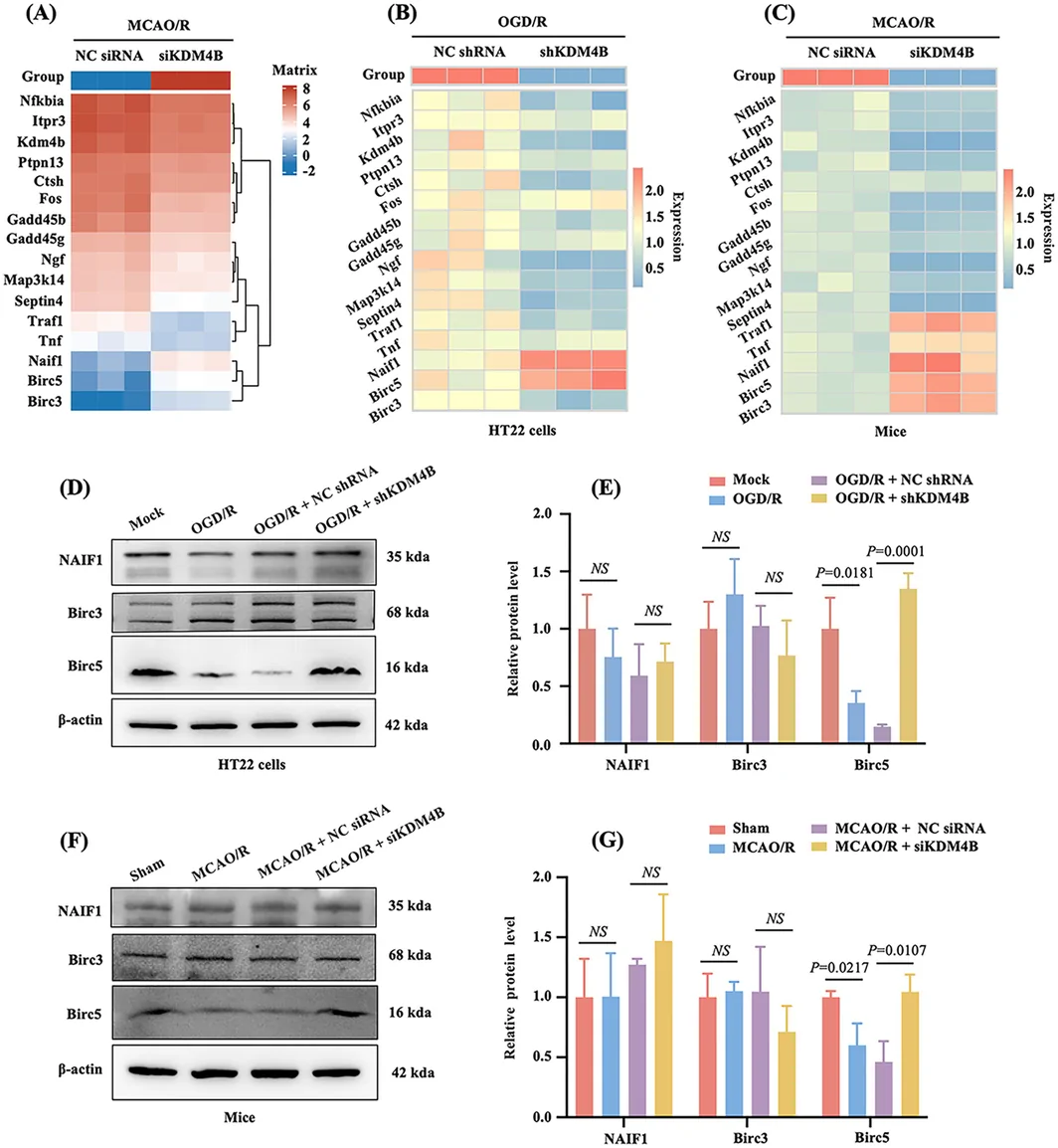
KDM4B knockdown reduces neuronal apoptosis associated with Birc5 upregulation. (A) Heatmap for differential transcriptional changes of the constitutive genes in the apoptotic pathway in KEGG enrichment analysis, including Nfkbia, Itpr3, Kdm4b, Ptpn13, Ctsh, Fos, Gadd45b, Gadd45g, Ngf, Map3k14, Septin4, Traf1, Tnf, Naif1, Birc5, and Birc3. *n* = 3 independent biological replicates per group. (B) Heatmap for the qPCR analysis of the constitutive genes in the apoptotic signaling pathway (including Nfkbia, Itpr3, Kdm4b, Ptpn13, Ctsh, Fos, Gadd45b, Gadd45g, Ngf, Map3k14, Septin4, Traf1, Tnf, Naif1, Birc5, and Birc3) in HT22 cells with or without KDM4B knockdown under OGD/R condition. *n* = 3 independent biological replicates per group. (C) Heatmap for the qPCR analysis of the constitutive genes in the apoptotic signaling pathway (including Nfkbia, Itpr3, Kdm4b, Ptpn13, Ctsh, Fos, Gadd45b, Gadd45g, Ngf, Map3k14, Septin4, Traf1, Tnf, Naif1, Birc5, and Birc3) in ischemic stroke mice with or without KDM4B knockdown. *n* = 3 mice per group. (D, E) The expressions and quantification of NAIF1, Birc3, and Birc5 in HT22 cells with or without KDM4B knockdown under OGD/R condition. *n* = 3 independent biological replicates per group. (F, G) The expressions and quantification of NAIF1, Birc3, and Birc5 in ischemic stroke mice with or without KDM4B knockdown. *n* = 3 mice per group. NS, no statistical difference. The data represent at least three independent experiments.

## 4 Discussion

In this study, we have elucidated the function and mechanism of lysine demethylase KDM4B on neuronal apoptosis and neurological impairment following ischemic stroke. Our results showed that KDM4B is primarily expressed in neurons, with a significant downregulation in the acute phase after ischemic stroke, followed by a gradual upregulation. Phenotypic experiments demonstrated that KDM4B knockdown reduced the expression of apoptosis‐related proteins while increasing the expression of anti‐apoptotic proteins, ultimately inhibiting neuronal apoptosis both in vitro and in vivo ischemic stroke models. Consequently, KDM4B knockdown not only significantly improved cerebral infarct volume, neurological deficit scores, cerebral tissue structural damage, and psychomotor dysfunction in ischemic stroke mice but also markedly alleviated cell injury in neurons subjected to OGD/R stimulation. Mechanistic studies suggested that KDM4B knockdown was associated with upregulation of Birc5 expression, a known target that plays a crucial role in inhibiting apoptotic cascades.

Histone methylation is a key biological process closely linked to cell growth and differentiation, normal organ development, and the onset and progression of various diseases [17]. The KDM4 (JMJD2, Jumonji domain‐containing protein 2) family consists of histone demethylases that catalyze the removal of methyl groups from lysine residues at the histone termini [10, 18]. As a prominent member of the KDM4 family, KDM4B has garnered significant attention due to its critical role in tumor pathogenesis. The full‐length KDM4B protein contains one JmjN domain, one JmjC domain, two PHD domains, and two Tudor domains [7]. The JmjC domain acts as the catalytic core of KDM4B, containing a binding site for divalent iron ions and driving histone demethylation. Upon binding to divalent iron ions, KDM4B, together with substrate cofactors such as α‐ketoglutarate and O_2_, catalyzes hydroxylation reactions, leading to the removal of dimethyl or trimethyl groups from terminal lysine residues on histones [7, 10]. KDM4B displays high affinity for the lysine residues H1.4K26, H3K9, H3K27, H3K36, and H4K20, and specifically catalyzes their demethylation, thereby regulating the transcriptional expression of target genes [7, 10]. Consequently, KDM4B has recently become an important therapeutic target in tumors, as it can regulate the transcription of various oncogenes through histone demethylation and participate in downstream carcinogenic signaling pathways. For example, KDM4B demethylates H3K36me3 modifications in the PHGDH (Phosphoglycerate dehydrogenase) promoter, which reduces PHGDH expression and thereby inhibits the proliferation, epithelial‐mesenchymal transition, metastasis, and stemness of breast cancer cells [19]. In clear cell renal cell carcinoma (ccRCC), KDM4B directly removes the repressive H3K9me3 modifications in the VEGFA (Vascular endothelial growth Factor A) promoter region to activate VEGFA transcription, ultimately sustaining bevacizumab resistance in ccRCC [20]. Beyond its role in regulating oncogene transcription, KDM4B has also been reported to physically interact with non‐histone oncoproteins. Depending on its demethylase activity, it modulates the functions of these proteins in carcinogenesis, thereby activating oncogenic signaling pathways. For instance, in colorectal cancer, KDM4B interacts with TRAF6 and enhances TRAF6‐mediated AKT ubiquitination, thereby activating the AKT signaling pathway and ultimately promoting glucose metabolism [8]. In castration‐resistant prostate cancer (CRPC), KDM4B partners with c‐Myc to form a complex and regulates the binding of c‐Myc to the promoters of metabolism‐related genes (LDHA, ENO1, and PFK) via its demethylase activity, thereby synergistically promoting c‐Myc‐mediated transactivation. Consequently, this reshapes the metabolic program of CRPC cells from the Warburg effect to oxidative phosphorylation, promoting CRPC proliferation and progression [9]. These studies indicate that KDM4B has emerged as an increasingly critical regulator of tumor‐related processes, including proliferation, metastasis, and drug resistance. Additionally, recent research has highlighted the crucial role of KDM4B in aging, obesity, and metabolic dysfunction [11, 12, 13, 21]. In the field of neurological disorders, our present study is the first to demonstrate the deleterious effect of KDM4B on neurological impairment following ischemic stroke. Specifically, KDM4B knockdown ameliorated cerebral infarction, cerebral tissue structural disruption, and sensorimotor dysfunction in the in vivo ischemic stroke model while alleviating neuronal injury in the in vitro model. Our current research expands the phenotypic role of KDM4B in non‐tumor diseases.

Apoptosis is a classic type of molecularly regulated cell death that can be triggered by physiological, pathological, or pathogenic stimuli, and its initiation can be categorized into two pathways: the intrinsic pathway and the extrinsic pathway. In intrinsic apoptosis, intracellular stressors such as nutrient deprivation and DNA damage block pro‐survival BCL‐2 family members (BCL‐2, BCL‐XL, MCL‐1), thereby releasing pro‐apoptotic proteins BAK and BAX to trigger mitochondrial outer membrane permeabilization and cytochrome c release. Cytochrome c in the cytoplasm binds to apoptotic protease‐activating factor 1 (APAF‐1) and drives its oligomerization into a heptameric structure. Subsequently, APAF‐1 interacts with the caspase recruitment domain (CARD) of caspase‐9 via CARD‐CARD homophilic interaction to form the apoptosome. This complex activates the initiator caspase‐9, which then cleaves and activates the effector caspases‐3, −6, and− 7, unleashing their apoptotic executioner functions [22]. In the extrinsic apoptosis pathway, extracellular signals—such as Fas ligand (FASL), tumor necrosis factor (TNF), or TNF‐related apoptosis‐inducing ligand (TRAIL)—are recognized by their respective death receptors on the plasma membrane. Upon ligand binding, the cytoplasmic domains of these death receptors interact with the adapter molecules FAS‐associated via death domain (FADD) and TNFRSF1A‐associated via death domain (TRADD), which recruit the initiator caspases‐8 and −10 to form the death‐inducing signaling complex (DISC). Following activation within the DISC, caspases‐8 and −10 cleave and activate effector caspases‐3, −6, and−7 to execute programmed cell death. Concurrently, activated caspase‐8 processes the BH3‐only protein BID into tBID (truncated BID), establishing a cross‐talk with the intrinsic apoptotic pathway [23]. It is evident that the intrinsic and extrinsic apoptotic pathways are both independent and interconnected, sharing common downstream components—effector caspases‐3, −6 and−7—which are crucial for cellular apoptosis. In the pathogenesis of ischemic stroke, multiple intracellular disruptive factors (e.g., calcium ions influx, calpain activation, reactive oxygen species, and DNA damage) are capable of activating the intrinsic apoptotic signaling pathway in neurons. Concurrently, the binding of death ligands to their cognate death receptors on the neuronal surface triggers extrinsic apoptosis. Thus, neuronal apoptosis constitutes the predominant mode of neuronal death in the ischemic penumbra and exerts a critical impact on neurological function impairment [24]. Birc5, also designated as survivin, is a core member of the inhibitor of apoptosis protein (IAP) family. Structurally, it comprises a BIR domain at the amino terminus and an extended α‐helical region at the carboxyl terminus, and serves as a pivotal inhibitor of apoptosis [25, 26]. Birc5 exerts its antiapoptotic effects through direct or indirect suppression of the caspase cascade, which mediates the initiation and execution of apoptotic processes. Specifically, via its conserved BIR domain, Birc5 can directly abrogate the activity of mitochondrial apoptosis‐inducing factor 1 (AIFM1), caspase‐9, and caspase‐3 [25, 26, 27]. Furthermore, it indirectly attenuates caspase activity by interacting with Smac/DIABLO, thereby neutralizing the inhibitory effect of Smac/DIABLO on IAP family proteins [27, 28].

In the present study, to elucidate the phenotypic role of KDM4B in ischemic stroke, we analyzed the biological functions and signaling pathways regulated by KDM4B using RNA sequencing data. Our results strongly suggest that KDM4B knockdown inhibits the apoptotic pathway and attenuates the apoptotic process. Subsequently, results from molecular biological experiments confirmed that KDM4B knockdown exerts an inhibitory effect on pro‐apoptotic regulatory proteins, apoptotic initiator caspases, apoptotic effector caspases, and cellular apoptotic status. These findings indicate that the neuroprotective effect of KDM4B knockdown in ischemic stroke is mediated through the inhibition of neuronal apoptosis. Furthermore, upon analyzing the constitutive genes within the apoptotic signaling pathway, we observed that KDM4B knockdown tended to selectively upregulate Birc5, a well‐documented and crucial anti‐apoptotic protein [29, 30, 31]. More importantly, molecular biological experiments further validated this selective and significant upregulatory effect of KDM4B knockdown on Birc5 in both animal models and cellular models of ischemic stroke. This finding also provides a clear explanation for the inhibitory effect of KDM4B knockdown on neuronal apoptosis. It should be noted that our study has not yet clarified the molecular mechanism by which KDM4B knockdown upregulates Birc5. In future studies, we will investigate the molecular cascade by which KDM4B regulates Birc5 expression based on its histone demethylase activity, and further validate the precise role of Birc5 in KDM4B‐mediated neuroprotection through intervention experiments targeting Birc5. Mechanistically, our data showed that KDM4B knockdown was associated with a selective increase in Birc5 expression, accompanied by reduced Bax expression, increased Bcl‐2 expression, and decreased activation of the caspase cascade. Given the established anti‐apoptotic function of Birc5/survivin, these findings suggest that Birc5 upregulation may contribute to the anti‐apoptotic effect of KDM4B knockdown. However, the present study does not establish whether KDM4B directly regulates Birc5 transcription through histone demethylation. Therefore, the KDM4B–Birc5 link should be interpreted as inferential at this stage. Future studies using ChIP‐qPCR or ChIP‐seq to examine KDM4B occupancy and histone methylation changes, such as H3K9me3, H3K36me3, or other relevant marks at the Birc5 promoter, are needed to define the precise epigenetic mechanism.

From a translational perspective, KDM4B may represent a potential therapeutic target for reducing neuronal apoptosis after ischemic stroke. However, important safety concerns should be considered. KDM4B is widely involved in epigenetic regulation [32], tumor progression [33], metabolism [34], and cell survival [35], while Birc5/survivin is a well‐known anti‐apoptotic protein frequently associated with malignancy [36]. Therefore, systemic or long‐term manipulation of the KDM4B–Birc5 axis may carry potential risks, including aberrant cell survival or tumor‐promoting effects. Future therapeutic strategies should focus on brain‐targeted, cell‐type‐specific, and temporally controlled modulation of KDM4B activity. In addition, long‐term safety assessment will be necessary before considering clinical translation.

Several limitations should be acknowledged. First, only male mice were used in this study to reduce variability associated with the estrous cycle and estrogen‐mediated neuroprotection during the acute phase of cerebral ischemia/reperfusion injury [37, 38]. However, sex is an important biological variable in stroke pathophysiology and treatment response. Therefore, whether KDM4B knockdown exerts similar protective effects in female animals remains to be determined. Second, although randomization, blinding, and predefined exclusion criteria were applied, detailed group‐specific records of animal exclusion, mortality, and attrition were not prospectively maintained for all experimental cohorts. Therefore, we were unable to provide a complete group‐by‐group attrition summary. Future studies will prospectively record and report animal allocation, exclusion, mortality, and final analysis numbers in full accordance with the ARRIVE guidelines. Although HT22 cells are widely used as neuronal‐like cells in ischemia‐related in vitro studies, they do not fully recapitulate the biological features of primary cortical neurons. Validation in primary neurons and other neural cell types would strengthen the translational relevance of our findings. In addition, our in vivo analyses were mainly performed at 48 h after reperfusion, representing an acute injury phase. Whether KDM4B knockdown provides sustained neuroprotection during the subacute and chronic phases remains unknown. Moreover, long‐term neurological outcomes, including cognitive function, sensorimotor recovery, brain atrophy, and survival, were not evaluated. Future studies incorporating longer reperfusion time points and comprehensive behavioral assessments are warranted.

## 5 Conclusion

Phenotypically, our study results confirm that KDM4B knockdown protects neurons, mitigates brain tissue damage, and ameliorates neurobehavioral impairments—ultimately exerting a neuroprotective effect in ischemic stroke. Mechanistic studies revealed that KDM4B knockdown was associated with the specific upregulation of Birc5, whose expression is known to be involved in the inhibition of apoptotic cascades.

## Author Contributions

J.Z. and Q.H. designed and supervised the whole project; S.D. performed the main experiments and analyzed the data; N.M. supplementally performed the experiments; Q.H. and S.D. wrote the manuscript and made the figures; M.J., K.H., and M.X. raised the animals, performed data analysis, and provided help for the revision experiments.

## Funding

This work was supported by the National Natural Science Foundation of China (82472230 and 82302474) and the Natural Science Foundation of Chongqing Science and Technology Committee (CSTB2025NSCQ‐GPX1243).

## Ethics Statement

All animal experiments in this study were approved by the Institutional Ethics Committee of Chongqing Medical University (Approval ID: IACUC‐CQMU‐2024‐0636) and were conducted in accordance with the ARRIVE guidelines.

## Conflicts of Interest

The authors declare no conflicts of interest.

## Supporting information


**Data S1:** Supporting Information.

## Data Availability

The raw RNA‐seq reads generated in this study were deposited in the NCBI Sequence Read Archive (SRA) under BioProject accession PRJNA1516130. This BioProject ID enables efficient dataset searching via the Entrez system. The dataset will be released immediately upon manuscript publication and is accessible at https://www.ncbi.nlm.nih.gov/sra/PRJNA1516130. All other original data that support the findings of this study are also available from the corresponding author upon reasonable request.
