# Supplementary material for: KDM4B Knockdown Attenuates Neurological Deficits in Ischemic Stroke by Inhibiting Neuronal Apoptosis Associated With Birc5 Upregulation
Source: CNS Neurosci Ther. 2026 Nov 11;32(11):e71264. doi: 10.1002/cns.71264 (PMC13752712; doi:10.1002/cns.71264)
Supplement: Supplementary file 1 [file CNS-32-e71264-s001.docx]

**KDM4B Knockdown Attenuates Neurological Deficits in Ischemic Stroke by Inhibiting Neuronal Apoptosis Associated with Birc5 Upregulation**

**Authors:**

Siyu Deng^1,2,*^, Nueraili Maihemuti^2,3,*^, Mengjie Jia^2,4^, Keli Huang^1,2^, Mao Xie^1,2^, Qi He^1,2,#^, Jing Zhao^1,2,#^

**Affiliations**

1. Department of Pathophysiology, Chongqing Medical University, Chongqing, 400010, People’s Republic of China;

2. Center for Neuroscience Research, Chongqing Medical University, Chongqing, 400010, People’s Republic of China;

3. Department of Pathology, Chongqing Medical University, Chongqing, 400010, People’s Republic of China;

4. Department of Critical Care Medicine, the First Affiliated Hospital of Chongqing Medical University, Chongqing, China;

* These authors contributed equally to this study.

\# Corresponding author: Qi He and Jing Zhao.

E-mail: [heqi191045@cqmu.edu.cn](mailto:heqi191045@cqmu.edu.cn) (Qi He); [zhaojing@cqmu.edu.cn](mailto:zhaojing@cqmu.edu.cn) (Jing Zhao);

**Materials and methods**

1. **Mice**

All animal experiments in this study were approved by the Institutional Ethics Committee of Chongqing Medical University (Approval ID: IACUC-CQMU-2024-0636) and were conducted in accordance with the ARRIVE guidelines. A total of 102 adult male C57BL/6J mice (18–22 g) were used. Animals were housed under a 12‑h light/dark cycle at 21–26 °C with free access to food and water.

Sample size was determined based on our preliminary experiments and previous studies using similar MCAO/R endpoints, considering expected effect size, variability, and animal welfare. Mice were randomly assigned to experimental groups using a computer‑generated randomization sequence, with group allocation performed before surgery by an investigator not involved in outcome assessment.

Animals were excluded according to predefined criteria: unsuccessful MCAO/R modeling, subarachnoid hemorrhage, absence of neurological deficit after MCAO, death before the scheduled endpoint, or insufficient cerebral blood flow reduction/reperfusion (assessed by laser Doppler flowmetry).

Due to the nature of the surgical procedure, the surgeon could not be fully blinded during MCAO/R surgery but was not involved in subsequent data acquisition or analysis. All investigators responsible for infarct volume quantification, histological staining, immunofluorescence/TUNEL quantification, and behavioral testing were blinded to group allocation.

2. **MCAO/R** **surgery and Laser Doppler flowmeter (LDF)**

The surgical procedure of middle cerebral artery occlusion and reperfusion (MCAO/R) in mice was performed as previously described [36-38]. Briefly, mice were intubated and kept under 3% isoflurane anesthesia. The mice were placed in the supine position, and a midline cervical incision was made. A nylon monofilament was advanced from the left external carotid artery to the ipsilateral middle cerebral artery to occlude cerebral blood flow for 1 hour. The nylon monofilament was then slowly withdrawn to initiate blood reperfusion for the indicated durations. Sham-operated mice were anesthetized and underwent the same surgical procedure without the insertion of the nylon monofilament.

The changes in regional cerebral blood flow during the MCAO/R procedure were monitored using a laser Doppler flowmeter (LDF). Briefly, mice were placed in a stereotaxic apparatus, and the bregma was exposed. The probe was then fixed at 0.3 mm posterior to the bregma and 1.0 mm left of the midline. A successful MCAO/R surgery was defined as a >85% reduction in blood flow during ischemia and a >70% recovery during reperfusion.

3. **Stereotaxic injection of siRNA**

KDM4B siRNA-1 (sense primer 5‐GCUGAAGGUGGAGAUCAAA‐3 and antisense primer 5‐ UUUGAUCUCCACCUUCAGC‐3), KDM4B siRNA-2 (sense primer 5‐GAAAGGACAUGGUCAAGAU‐3 and antisense primer 5‐AUCUUGACCAUGUCCUUUC‐3), and KDM4B siRNA-3 (sense primer 5‐GCGCAGACAAAUUAGUGUGAA‐3 and antisense primer 5‐UUCACACUAAUUUGUCUGCGC‐3), were chemically synthesized by Tsingke Biotechnology. The siRNA was dissolved with RNase‐free water and then stereotaxically injected into the left lateral ventricle (coordinates: AP -0.3 mm, ML -1.0 mm, DV -2.5 mm) and paracortical regions (coordinates: AP +0.3 mm, ML ±2.1 mm, DV -2.2 mm). The injection parameters were as follows: 10 µL/OD per mouse at a rate of 0.5 µL/min. The needle was retained for 10 minutes to prevent reflux.

4. **Infarct volume quantification**

Mice were euthanized under deep anesthesia. The brains were rapidly harvested, snap-frozen in liquid nitrogen, and coronally sectioned into five slices (2 mm thick) using a rodent brain matrix. The brain sections were immersed in 2% TTC solution for 20 minutes at 37°C in the dark to visualize viable tissue (red staining), followed by fixation in 4% paraformaldehyde solution for 48 hours at room temperature. Digital images of the brain sections were captured with a digital camera and analyzed using ImageJ software (Version 8.0). To avoid edema-induced hemispheric expansion, the infarct volume was calculated as the percentage of the non-infarcted tissue volume in the ipsilateral hemisphere relative to that in the contralateral hemisphere.

5. **mNSS (modified Neurological Severity Score)**

The degree of neurological deficit in experimental mice was blindly assessed by two investigators using a standardized 6-point scale: 0 (no neurological deficit), 1 (contralateral forelimb retraction during tail suspension), 2 (spontaneous contralateral circling), 3 (lateral rolling during ambulation), 4 (akinesia), and 5 (mortality). Mice with scores of 0 or 5 were excluded to minimize non-ischemic confounders.

6. **Hematoxylin and eosin (H&E) and Nissl staining**

Mice were euthanized under deep anesthesia. The brains were rapidly harvested, fixed in 4% paraformaldehyde solution, dehydrated, paraffin-embedded, and coronally sectioned into 2 μm-thick slices. The sections were subjected to HE and Nissl staining according to the operating manual.

**7. Behavioral tests**

**7.1. Open Field Test**

Locomotor activity of experimental mice was evaluated using the open field test. Briefly, each mouse was individually placed in the central zone of a square arena, and the total distance traveled was recorded for 5 minutes using an automated tracking system. The arena was cleaned with 70% ethanol between trials to eliminate residual olfactory cues.

**7.2. Rotarod Test**

The motor coordination of experimental mice was evaluated using an accelerating rotarod apparatus as previously described [37, 38]. Briefly, each mouse was placed on the rotating rod, which accelerated from 4 to 40 rpm over 5 minutes, and subjected to three consecutive trials. A 15-minute rest interval was allowed between each trial. The average latency to fall from the rotating rod was recorded for each mouse.

**7.3. Grip Strength Test**

Both the bilateral forelimb strength and the bilateral limbs strength were quantified using a digital grip strength device [37, 38]. Briefly, each mouse was placed on a horizontal metal grid, and its bilateral forelimbs or all four limbs were allowed to firmly grasp the probe of the grip strength meter. Peak force was recorded during horizontal tail traction. Each mouse was tested five times with a 1.0-minute interval between trials. Mice exhibiting excessive struggling were retested after 15 minutes. Motor muscle strength was expressed as the average of the three highest peak force values.

8. **Cell culture and the oxygen-glucose deprivation/reoxygenation (OGD/R) model**

The mouse HT22 neuronal cells were kept in our laboratory and routinely cultured in DMEM medium (C11995500BT, Gibco, USA) supplemented with 10% fetal bovine serum (viva cell, C04001-500) and 1% penicillin-streptomycin (15070063, Gibco, USA) in 5% CO_2_ at 37℃.

The procedure of the oxygen-glucose deprivation/reoxygenation model has been described in our previous study [37, 38]. Briefly, HT22 cells were subjected to glucose-free DMEM medium in a hypoxic incubator (1% O_2_, 5% CO_2_, and 94% N_2_) at 37 °C for 4 h. Subsequently, HT22 cells were cultured in normal DMEM medium containing 10% fetal bovine serum and 1% penicillin-streptomycin with reoxygenation for 48 h.

9. **Stable knockdown of KDM4B in HT22 cells**

Stable knockdown of KDM4B in HT22 cells was achieved using a lentiviral system. Briefly, wildtype HT22 cells were infected with lentiviral particles encoding KDM4B-specific short hairpin RNA (shKDM4B: GAAAGGACAUGGUCAAGAU). Stable polyclonal cell populations were selected using culture medium containing 4 μg/mL puromycin for 7 days, and the efficiency of KDM4B knockdown was validated by western blot analysis. HT22 cells infected with empty lentiviral particles (shControl) served as the control group.

10. **Lactate dehydrogenase (LDH) assay**

The positive control (maximum LDH release) was established by adding 10 μL of LDH release reagent to the control wells 1 hour prior to detection. A total of 120 μL of culture medium was collected from each well and transferred to a new 96-well plate. The medium was mixed with 60 μL of LDH detection working solution (Beyotime, China) and incubated in a dark incubator at 25°C for 30 minutes. Absorbance was measured at 490 nm using a microplate reader (Thermo, USA). Cell injury was expressed as the percentage of absorbance relative to that of the positive control.

11. **Flow cytometry of apoptosis**

HT22 cells were washed once with PBS and trypsinized with 0.025% EDTA-free trypsin solution. The cells were then incubated with Annexin V-FITC and propidium iodide before being analyzed by flow cytometry. The apoptotic rate was calculated as the sum of early and late apoptotic cells.

12. **Propidium Iodide (PI) staining**

The procedure for PI staining of cultured cells has been described in our previous study [37, 38]. Briefly, HT22 cells were seeded in 12-well plates and exposed to OGD/R treatment. The culture medium was removed, and the cells in each well were incubated with 500 μL of PI working solution (Beyotime, China) in an incubator for 30 minutes. PI stains dead cells with red fluorescence. Fluorescent images were captured using an upright fluorescence microscope (Leica DM3000, Germany). The HT22 cell death rate was calculated based on the fluorescent staining results. For PI staining quantification, 3 non-overlapping fields were randomly captured per well under identical imaging settings. PI-positive cells and total cells were counted using ImageJ by an investigator blinded to group allocation. The percentage of dead cells was calculated as the number of PI-positive cells divided by the total number of cells in the same field.

13. **Immunofluorescence staining**

Brain sections were incubated with primary antibodies overnight at 4°C. The primary antibodies used for immunofluorescence staining, including those against KDM4B, NeuN, GFAP, and Iba1, are listed in Table 2. After incubation, the sections were washed three times with PBST and then incubated with fluorescent secondary antibodies for 2 h at room temperature. Finally, the sections were counterstained with DAPI for 5 min at room temperature. Fluorescent images were captured using an upright fluorescence microscope (Leica DM3000, Germany).

14. **TUNEL staining**

Brain sections or cell coverslips were fixed with 4% paraformaldehyde solution, permeabilized with 0.1% Triton X-100, and incubated with TUNEL reaction mixture (Beyotime, China) in the dark. Nuclei were counterstained with DAPI (Beyotime, China). Stained images were captured using an upright fluorescence microscope (Leica DM3000, Germany) and quantified in three random fields per sample using ImageJ software. For TUNEL quantification in brain tissues, sections were collected from comparable coronal levels containing the peri-infarct cortex. For each animal, 5 sections were analyzed, and 3 non-overlapping fields in the peri-infarct region were imaged per section under identical microscope settings. For TUNEL quantification in HT22 cells, cells were randomly imaged in 5 non-overlapping fields per well, and 3 wells were analyzed for each independent experiment. TUNEL-positive nuclei and total nuclei (DAPI-positive cells) were counted using ImageJ manually by an investigator blinded to group allocation. The proportion of apoptotic cells was calculated as the percentage of TUNEL-positive cells relative to the total number of DAPI-positive cells in each field.

15. **Western blot analysis**

The cerebral cortex and cell pellets were lysed in RIPA lysis buffer and denatured with 2× SDS-PAGE loading buffer. Equal amounts of protein lysates were separated by SDS-PAGE and electrotransferred onto PVDF membranes. The membranes were blocked with 5% bovine serum albumin for 90 min at room temperature and then incubated overnight at 4°C with primary antibodies against KDM4B, Bax, Bcl-2, caspase-3, caspase-6, caspase-7, caspase-8, caspase-9, NAIF1, Birc3/cIAP2, Birc5, and β-actin. Detailed antibody information is provided in Table 2. After washing, HRP-conjugated secondary antibodies were applied to the membranes for 90 min at room temperature. Protein bands were visualized using enhanced chemiluminescence (Yeasen, China) and quantified densitometrically using ImageJ software. For each sample, the integrated density of the target protein band was measured after background subtraction and normalized by β-actin. Data were further normalized to the mean value of the control group for comparison across independent experiments.

16. **qPCR assay**

The procedures for total RNA isolation, cDNA synthesis and gene amplification in this study were similar to our previously published research [37]. Data were normalized to β-actin expression, and the relative fold change was calculated using the 2^⁻ΔΔCt^ method. The primer sequences used in the qPCR assay are provided in Table 1.

17. **RNA‑seq analysis**

Total RNA was extracted from the cerebral cortical penumbra of the sham group, MCAO + NC siRNA group, and MCAO + KDM4B siRNA group (n = 3 per group), and preserved in TRIzol reagent. RNA quality control, library construction, and sequencing were performed by Sino Science Life Technology Co., Ltd. (Shanghai, China) using the Illumina NovaSeq platform. The raw sequencing data were processed and analyzed for differential gene expression. The raw RNA‑seq reads have been deposited in the NCBI Sequence Read Archive (SRA) under BioProject accession PRJNA1516130, which enables dataset retrieval via the Entrez system.

18. **Statistical Analysis**

Statistical analyses were performed using IBM SPSS Statistics 22.0, and statistical drawings were constructed by GraphPad Prism 9.0. Data normality was assessed using the Shapiro–Wilk test. Results following a normal distribution are expressed as Mean±SD. For comparisons among multiple groups, one-way ANOVA followed by Tukey’s post-hoc analysis was applied. Between-group differences, assuming homoscedasticity, were evaluated using Student’s t-test. Non-parametric data were subjected to Kruskal–Wallis analysis. A *P*-value <0.05 was considered statistically significant. For in vivo experiments, n indicates the number of mice per group. For in vitro experiments, n indicates the number of independent biological replicates (each replicate represents an independent cell culture preparation).

**References**

36.	Huang X, Tan J, Ji Y, Luo J, Zhao Y, Zhao J. *BRCC3 mediates inflammation and pyroptosis in cerebral ischemia/reperfusion injury by activating the NLRP6 inflammasome*. CNS Neurosci Ther. 2024 Mar;30(3):e14697.

37. He Q, Meng C, Jia M, Tan J, Huang K, Gan H, Li L, Zhao J. *NLRP6 deficiency inhibits neuroinflammation and ameliorates brain injury in ischemic stroke by blocking NLRs inflammasomes activation through proteasomal degradation of pro-caspase-1*. Neurobiol Dis. 2024 Mar;192:106434.

38. Tan J, Wang M, Huang K, Xie M, Zheng Y, Luo J, Tang J, Zhai X, He Q, Zhao J. *Phosphorylation of NLRP6 at Serine 27 site is critical for NLRP6 inflammasome activation and neurological impairment in ischemic stroke*. Cell Commun Signal. 2026 Jan 28;24(1):128.
